# Monitoring Involuntary Muscle Activity in Acute Patients with Upper Motor Neuron Lesion by Wearable Sensors: A Feasibility Study

**DOI:** 10.3390/s21093120

**Published:** 2021-04-30

**Authors:** Andrea Merlo, Maria Giulia Montecchi, Francesco Lombardi, Xhejsi Vata, Aurora Musi, Mirco Lusuardi, Roberto Merletti, Isabella Campanini

**Affiliations:** 1 LAM-Motion Analysis Laboratory, S. Sebastiano Hospital, Neuromotor and Rehabilitation Department, Azienda USL-IRCCS di Reggio Emilia, Via Circondaria 29, 42015 Correggio, Italy; xhejsivata@yahoo.it (X.V.); aurora.musi@gmail.com (A.M.); Isabella.Campanini@ausl.re.it (I.C.); 2 Merlo Bioengineering, 43121 Parma, Italy; 3 Neurorehabilitation Unit, S. Sebastiano Hospital, Neuromotor and Rehabilitation Department, Azienda USL-IRCCS di Reggio Emilia, Via Circondaria 29, 42015 Correggio, Italy; Mariagiulia.Montecchi@ausl.re.it (M.G.M.); Francesco.Lombardi@ausl.re.it (F.L.); 4 Neuromotor and Rehabilitation Department, Azienda USL-IRCCS di Reggio Emilia, Via Circondaria 29, 42015 Correggio, Italy; Mirco.Lusuardi@ausl.re.it; 5 Laboratory for Engineering of the Neuromuscular System (LISiN), Department of Electronic Engineering and Telecommunications, Politecnico di Torino, 10129 Turin, Italy; roberto@robertomerletti.it

**Keywords:** wearable sensors, surface EMG, upper motor neuron lesion, long-lasting acquisitions, involuntary muscle activity, spastic dystonia, muscle overactivity

## Abstract

Sustained involuntary muscle activity (IMA) is a highly disabling and not completely understood phenomenon that occurs after a central nervous system lesion. We tested the feasibility of in-field IMA measuring at an acute rehabilitation ward. We used wearable probes for single differential surface EMG (sEMG), inclusive of a 3D accelerometer, onboard memory and remote control. We collected 429 h of data from the biceps brachii of 10 patients with arm plegia. Data quality was first verified in the time and frequency domains. Next, IMA was automatically identified based on the steady presence of motor unit action potential (MUAP) trains at rest. Feasibility was excellent in terms of prep time and burden to the clinical staff. A total of 350.5 h of data (81.7%) were reliable. IMA was found in 85.9 h (25%). This was often present in the form of exceedingly long-lasting trains of one or a few MUAPs, with differences among patients and variability, both within and between days in terms of IMA duration, root mean square (RMS) and peak-to-peak amplitude. Our results proved the feasibility of using wearable probes for single differential sEMG to identify and quantify IMA in plegic muscles of bedridden acute neurological patients. Our results also suggest the need for long-lasting acquisitions to properly characterize IMA. The possibility of easily assessing IMA in acute inpatients can have a huge impact on the management of their postures, physiotherapy and treatments.

## 1. Introduction

Spastic dystonia is one of several types of muscle overactivity that can develop in patients after an upper motor neuron lesion (UMNL), as a result of pathological processes involving the motor areas of the brain or their connections to the spinal cord, such as cerebrovascular accidents (stroke), traumatic brain injuries, and post-anoxic coma [1]. Spastic dystonia is defined as a spontaneous muscle activation occurring at rest, in the absence of any phasic stretch or any voluntary command [2,3,4,5]. According to the literature, spastic dystonia contributes to the development of acquired deformities, the alteration of joint mobility caused by an increased resistance of the muscle–tendon unit to its stretch, and it increases the disability of the patient [4,5,6].

Spastic dystonia is characterized by sustained involuntary muscle activity (IMA) [7,8,9]. The pathophysiological mechanisms underlying IMA are still unknown [5,8,10]. IMA may determine abnormal joint postures: this facilitates soft tissue rearrangement and the development of muscle shortening [6,11,12]. These events inevitably lead to incorrect postures, facilitating and augmenting the phenomenon of overactivity as in a vicious circle [6].

In clinical practice, the assessment of overactivity is typically carried out by means of clinical scales such as the Modified Ashworth Scale and the Tardieu Scale. These scales are used in detecting spasticity, i.e., the presence of excessive reflexes detected by an assessor who applies a fast stretch to a muscle. The Modified Ashworth Scale uses a five-level grading classification to score the resistance perceived by the operator during joint mobilization at high velocity [13]. The Modified Tardieu Scale reports the difference between the angle of muscle reaction measured during a fast passive stretch and the passive range of motion measured during a slow passive stretch [13]. However, these scales can neither be used to monitor the presence of all forms of overactivity as IMA, nor to detect IMA insurgence including tracking its variation during the daytime and its progression during hospitalization. Early access to information on the insurgence of IMA and its characteristics may lead to changes in the evaluation of overactivity, in a patient’s rehabilitation, physiotherapy and/or in their pharmacological treatment [14].

IMA can be recorded using surface EMG (sEMG) [7,8,9,10]. Early studies in the 1960s demonstrated the possibility of visually identifying motor units in sEMG, with bipolar electrodes, during low level contractions [15]. In the last 20 years, High Density sEMG (HDsEMG) systems, based on two-dimensional electrode grids, and signal processing algorithms have been developed to decompose sEMG into its constituent trains of motor unit action potentials (MUAPT). This literature has been mainly published in technical journals and is not easily accessible for clinicians. Efforts are under way to overcome this issue by divulging the information and transferring technology [16,17,18].

Campanini and colleagues conducted the assessment of IMA by sEMG in a study using high-density surface electrode arrays. In their work, focused on acute patients admitted to a ward for acute rehabilitation immediately after their discharge from the stroke unit, measurements of the biceps brachii (BB) muscle lasting no more than five minutes were carried out twice a day, three times a week for four weeks while patients were lying in their bed [7]. On the one hand, this study proved the possibility of identifying and quantifying IMA in these patients by using high-density sEMG (HDsEMG). On the other hand, the variability found among measurements highlighted the need for long-lasting acquisition that covers the whole day, along with the need for a minimally invasive instrumental setup with respect to the clinical routine (nursing, physiotherapy), patients’ postures (lying in bed, sitting on a wheelchair) and movement of the analyzed limb.

The availability of miniaturized wearable probes for bipolar sEMG recordings, with onboard memory, inclusive of inertial sensors, could provide a compromise satisfying both these needs. These probes allow for hours of continuous data acquisition without any burden to either the patient or the hospital staff. These probes were not initially designed to be used on patients in critical condition. Their ability to withstand electromagnetic interferences (EMIs) produced by motors or pumps present in the supporting devices located near the patient’s bed has never been tested. EMI sources may include pumps, as in the case of percutaneous endoscopic gastrostomy (PEG) used to supplement nutrition and hydration, tracheostomy vacuum cleaners, compressors of anti-decubitus air mattresses, and the electric motors in motorized beds and lifters. Large motion artifacts could also corrupt the data during patient handling (from bed to wheelchair) and during physiotherapy and joint mobilization at the bedside.

The aim of this study was to evaluate the feasibility of assessing IMA duration and its characteristics in patients admitted to an acute rehabilitation ward, and receiving everyday clinical activities, by means of sEMG wearable probes in a single differential configuration.

## 2. Materials and Methods

### 2.1. Patient Selection

This was a feasibility study that did not affect the clinical and rehabilitative pathway of the included patients in any way.

Patients hospitalized in an acute rehabilitation ward at our institution over a six-month period were included according to the following criteria: UMNL of any etiology, upper limb (UL) plegia, i.e., complete UL paralysis (absence of any voluntary movement at the affected upper limb, including fingers) to make sure to record only IMA.

Patients were excluded in the case of hydrocephalus, behavioral problems subsequent to the lesion, as assessed by a score ≥ 4 of the Level Cognitive Functional Scale, isolation due to infections, including HIV, known hypersensitivity to electrode gel or glue. 

The study was approved by the local Ethical Committee (2015/0015247). Informed consent was obtained before enrollment from either the patient or their legal guardian as per Italian law.

Throughout the study, patients were pseudo-anonymized using an alphanumeric ID (e.g., 007-U). Both arm sides were also included for patients when bilateral plegia was present, e.g., 001-Br (right); 001-Bl (left).

### 2.2. Definitions Used in This Study

In this study, we will use the following definitions:

IMA: presence of BB muscle activity without any UL acceleration (see below), with the patient at rest.

sEMG activity: any presence of muscle activity (including IMA) during the recording, irrespective of the amount of UL acceleration recorded by the probe, i.e., with the patient at rest, during passive mobilization, during posture changes (e.g., from supine to sitting) and also during nursing or physiotherapy activities. 

### 2.3. Protocol and Data Acquisition Procedures

**Figure 1:**
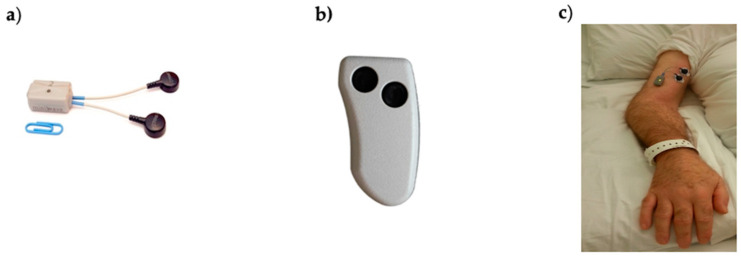
(**a**) Cometa Mini Wave Infinity wearable sEMG probe with embedded accelerometer and onboard memory used in this study; (**b**) remote control used to activate/deactivate probes; (**c**) electrodes and probe placed on the biceps brachii of one study subject.

Data acquisition was performed by wearable probes (Mini Wave Plus, Cometa, Italy) commonly used for dynamic sEMG acquisition, such as gait analysis or performance analysis in sports. These are part of a commercial system for surface electromyography consisting of eight (or sixteen) probes that can either transmit data to a receiving unit connected to a PC for real-time usage or store them onboard. The model we used was Mini Wave Infinity (18.5 × 32.5 × 12.6 mm) weighing 10 g (Figure 1). This probe allowed for the acquisition of bipolar sEMG data and 3D acceleration at a sample frequency of 2000 Hz. Other features were bandwidth 10–500 Hz, input referred noise of 3.5 μV_RMS_, input impedance 20 MΩ at 50 Hz and A/D conversion on 16 bits.

The presence of an embedded accelerometer allows for the identification of periods without movement and periods with passive movements, which are usually a result of physiotherapy, joint mobilization, nursing activities, or the patient’s involuntary reflex movements or voluntary body movements (e.g., change in position). The probe is provided with an onboard memory, where data can be stored continuously for up to 8 h. Data recording was activated by pressing a small remote control (Figure 1).

Before each acquisition, a preliminary set-up of the probes was carried out at the motion analysis laboratory of our institution. This simply consisted of the association of the proper label (the patient ID) with each probe. Next, the probes were brought in the ward and applied on the BB of patients. The time needed for patient prepping was recorded in the activity diary of the study, as described in more detail below.

For each patient, BB of the affected UL(s) was assessed at least once a week. The acquisition period lasted until the appearance of trace movement at the upper limb, until the patient was transferred to a different ward, or at the end of the study period.

During the acquisition days, the patients’ routine was not affected. Standard ward activities, including medications, nursing and rehabilitation activities, as well as eating, resting or sleeping, were not altered. Recordings were scheduled in agreement with the ward staff, in order to avoid the days when the patient was bathed with the bath stretcher and the days when patients accessed the rehabilitative pool.

In this study, we used circular disposable Ag/AgCl electrodes with a hydrogel conductive paste, a round foam edge (Arbo-Kendall H124, with a 16 mm in diameter contact area), and a center-to-center inter-electrode distance of 25 mm. These dimensions imply spatial filtering that is not critical for this application [19].

During the first day after enrollment, a single electrode pair was applied to the BB and periodically monitored over a six-hour period to test for the subject’s possible hypersensitivity to the electrode gel and glue.

For all acquisitions, electrodes were placed on the BB according to SENIAM recommendations [20,21]. The probe was fixed to the arm’s skin with double-sided adhesive patches (provided by the manufacturer). No further probe fastening was adopted. Probes were activated using a remote control and left in place for about six hours between 8 a.m. and 2 p.m.

At the end of the recordings, probes were removed from all patients and sanitized according to standard ward protocol. Finally, data were downloaded to a PC and split in 5-min time segments, which meant about 70 files per muscle per day. Files were named according to the acquisition date and progressive file number. sEMG recordings were labeled using patient IDs.

All measurement-related procedures were carried out by two physiotherapists (PTs). They recorded patient prepping and the patient’s condition during the recording period (lying in bed, sitting on a wheelchair, nursing activities, physiotherapy, etc.) in a diary. Consequently, for each of the 5-min lasting data files, the corresponding patient status was also tracked. 

In line with the aim of this study, the in-field feasibility of the measurements was evaluated in terms of:

- Time needed for prepping;
- Lost measurements due to incompatibility with other clinical activities such as nursing and physiotherapy;
- Lost measurements due to technical problems or low data quality.

The first two were obtained based on the information recorded in the diary. The third was computed as the percentage of unreliable data segments over total data collection. 

The feasibility of IMA assessment was determined based on:

- The possibility of detecting motor unit action potentials in the recorded data;
- The possibility of quantifying IMA in terms of amplitude and duration;
- The possibility of monitoring IMA variations within a day and in different days.

### 2.4. sEMG Data Filtering 

Surface EMG data were bandpass filtered in the range 20–400 Hz to reduce noise [22]. A series of notch filters were iteratively applied to remove power line interferences and other EMIs, when present (a notch filter is a narrow band-reject filter used to remove powerline interference. The rejected bandwidth was 4 Hz, centered on the frequency to be removed). These were identified when a narrow peak was present in the power spectrum of the signal. The central stop frequency of the notch filter was then tuned to the frequency of the peak. Finally, in-band baseline noise was removed using an efficient wavelet denoising algorithm [23].

### 2.5. sEMG Analysis Procedures

An automatic two-step procedure was developed and applied to the filtered data. It consisted of a set of data quality controls followed by the identification of the presence of motor unit action potentials (MUAPs), either separated in MUAP trains or summed up in an interferential signal. 

Consecutive epochs lasting 10 s were analyzed. Each epoch was classified as containing unreliable data if at least one of the following exclusion conditions was met:

- Number of EMI-related harmonics ≥ 5, in the frequency domain;
- Epoch peak-to-peak amplitude range > 2000 mV, in the time domain.

Each epoch was classified as containing only baseline noise and no sEMG if at least one of the following conditions was met:

- Epoch peak-to-peak amplitude range < 50 mV, in the time domain;
- Epoch minimum spectral peak frequency < 25 Hz, in the frequency domain;
- Epoch minimum spectral mean frequency < 30 Hz, in the frequency domain.

The upper amplitude threshold was selected to discard data with large motion artifacts or with detached electrodes (that lead to amplifier saturation); the lower amplitude threshold was selected to minimize the risk of false-positive identification, i.e., the risk of classifying as MUAPs (see below) events presenting an MUAP-like morphology but barely overcoming the baseline noise. Thresholds in the frequency domain were selected to include only epochs with spectral characteristics similar to those of an EMG signal, either interferential or composed of a train of single motor units from deep sources. All the above thresholds were tuned based on a preliminary analysis of >500 epochs. 

The presence of muscle activity was assessed using the method described by Merlo et al. [24]. This method uses a wavelet-based enhancement of the matched filter technique to identify the presence of each MUAP-like shape in the signal. The presence of a rhythmic sequence of MUAPs with a minimum physiological firing rate was later verified. For this study, the minimum firing rate threshold was set at 4 pulses per second. Only when this condition was met, the signal segment containing rhythmic or interferential MUAPs was classified as muscle activity [24]. For each epoch, we computed the duration of muscle activity. This procedure provided information on muscle activation during each 10-s epoch, with a data reduction ratio of 20,000:1.

Finally, the duration of the EMG activity was saved, together with the RMS and peak-to-peak amplitudes over the onset period including the mean and the peak frequencies of the power spectrum.

### 2.6. Three-Dimensional Acceleration Analysis Procedures

The magnitude of the acceleration vector was low-pass filtered at 6 Hz. The choice of this low-pass cut-off frequency was based on experimental testing to remove the baseline noise without filtering out the acceleration resulting from physiotherapy sessions, including rhythmic UL mobilization. For each 10 s-lasting epoch, the presence of arm movement (yes/no) was detected when the variation in the modulus of the acceleration signal was higher than the set threshold of 30 × 10^−3^ g. This was set based on the study by Hurter and colleagues [25] and verified by means of preliminary acquisitions. Knowing if the arm was being moved or not allowed us to differentiate between IMA and other types of muscle overactivity. Typical situations when passive limb movement is expected are: physiotherapy sessions at the patient’s bedside (e.g., passive arm mobilization), during the patient’s transfers from bed to wheelchair, and during nursing activities. Large accelerations are expected during passive movement due to external interventions. Other possible sources of limb acceleration are voluntary trunk movements (e.g., subjects able to shift in bed), and involuntary movements of the limb elicited by pain or other reflexes.

## 3. Results

### 3.1. Sample

Ten subjects, five males and five females, age range 50–71 were included in the study. Patients had UMNL subsequent to ischemic stroke (three cases), hemorrhagic stroke (four cases) and post-anoxic coma (three cases), time from lesion ranging between 28 and 132 days, and UL plegia without any voluntary movement of the whole upper limb including fingers. Four patients had bilateral involvement, thus leading to 14 BB muscles being analyzed.

The patient enrollment period in the study varied between 2 and 8 weeks. The main cause for patient dropout was the appearance of trace movements of the UL. The number of patients simultaneously assessed in any given day varied between 1 and 5.

The time needed for patient prepping, as recorded in the activity diary, was less than 5 min per patient. The time required for the preliminary setup of the probes, where each probe was associated with a specific patient ID, was also limited to a few minutes.

In total, 429 h of data were recorded while patients received the necessary care and treatments (see Table 1). The median duration of the recordings was 5.9 h. In two cases, the extent of the acquisition was limited to less than two hours due to insufficient battery duration as a result of incorrect charging. No other technical problems arose during the study.

No patients reported any discomfort, and no changes were made to the ward’s activity because of the presence of the probe. Clinical staff, including MDs, PTs and nurses, did not report any problems in the patients’ management either.

The number of measurements lost due to incompatibility with other clinical activities was limited to two. Specifically, two acquisitions pertaining to one patient were put on hold at the request of the neurologist due to the patient’s critical condition.

### 3.2. Feasibility of the Measurement 

Measurement feasibility was verified for all the 154,440, 10-s lasting epochs (154,440 epochs × 10 s/epoch/3600 s/h = 429 h). Of these, 18.3% of epochs were corrupted by either EMIs (≥5 harmonics) or motion artifacts (peak-to-peak amplitude range > 2000 mV). Consequently, data were reliable in 81.7% of the whole acquisitions pool (350.5 h of good data).

**Figure 2:**
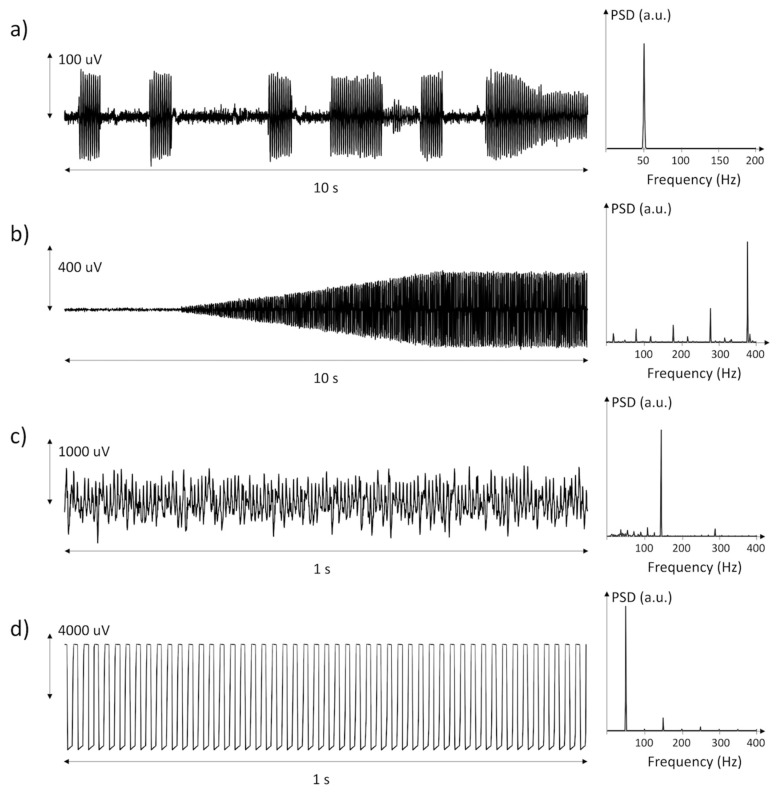
Three examples (**a**–**c**) of power line interference generated by disturbing equipment or loss of electrode–skin contact and one example (**d**) of saturation of the amplifier likely due to loss of electrode–skin contact.

Most of the discarded epochs (77%) were corrupted by EMI. These were periodic signals, with five or more harmonic peaks in the spectrum. Figure 2 shows a selection of power line interferences recorded in the data, along with their power spectral density. These interferences were generated by motors and pumps positioned close to the patient, by power line interference during patient handling, treatment (e.g., physiotherapy), or weakening of the adhesion in one or both electrodes. The occurrence of EMI was largely variable both between subjects and within individual measurement days.

**Figure 3:**
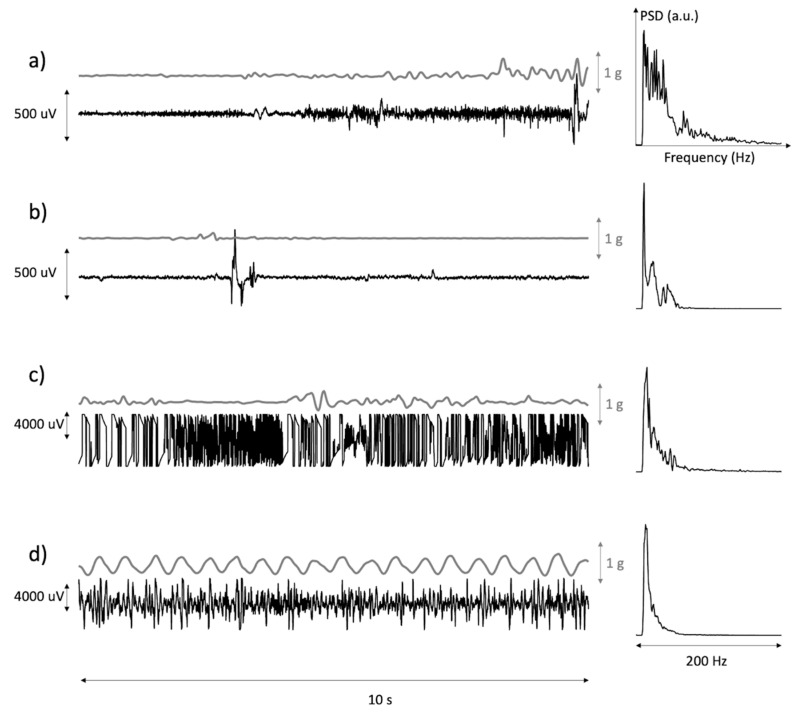
Time plots of the modulus of the acceleration of the EMG sensor and of the associated sEMG with its power spectrum. (**a**,**b**) are examples of occasional movement artifacts. (**c**) is an example of loss of electrode–skin contact(s) during movement. (**d**) is an example of passive movement of the arm during treatment.

The remaining discarded epochs (23%) presented characteristics attributable to either large-scale movement artifacts or electrode detachment leading to amplifier saturation. Figure 3 describes a selection of motion artifacts found in the data, together with their power spectral density and with the modulus of arm acceleration. These occurred during (a) muscle activation, (b) accidentally bumping into or coming in contact with the electrodes, (c) partial detachment of an electrode and (d) handling of the patient’s arm as in the case of physiotherapy.

### 3.3. Feasibility of sEMG Detection 

**Figure 4:**
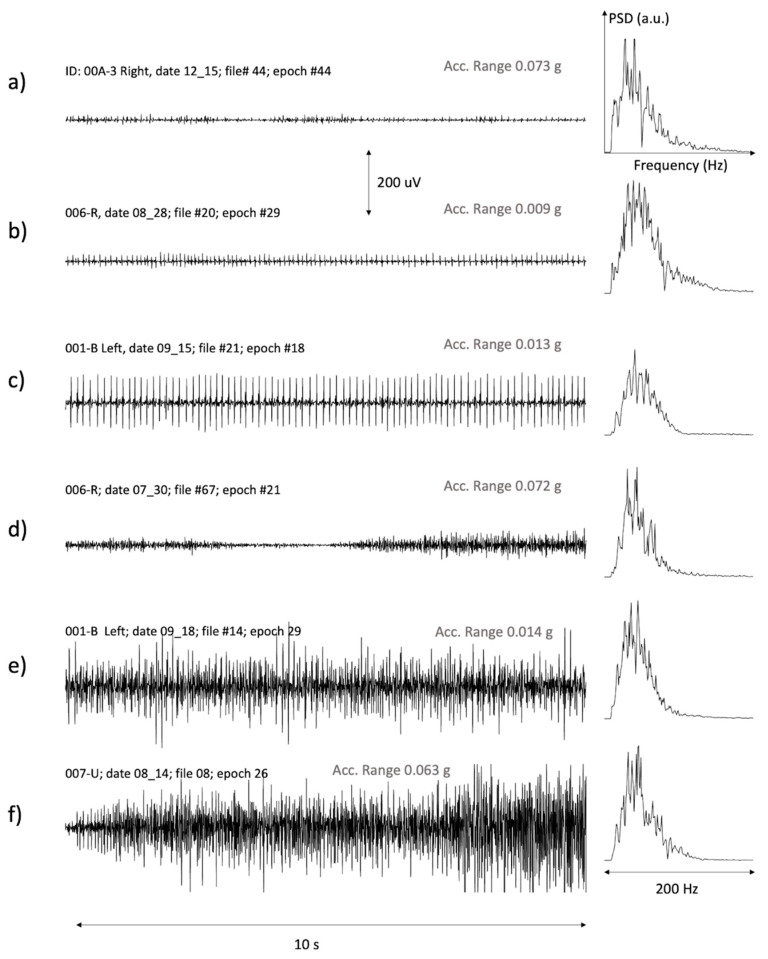
A representative example of good signals from different subjects with: (**a**,**b**) a few small motor units, (**c**) a large or very superficial motor unit active for a long time, (**d**) variable activation levels with muscle derecruitment followed by further recruitment, (**e**,**f**) interferential signals involving many motor units.

Figure 4 presents a selection of epochs with muscle activity. The power spectral density (PSD) of each signal is also shown. Each PSD plot is normalized with respect to the peak value.

The variability in sEMG characteristics is evident in Figure 4. We observed trains of single or a few MUs of different amplitudes. Examples are given in Figure 4a–c. We also observed epochs with MU recruitment and derecruitment, as shown in (d). Finally, we observed periods with prolonged interferential activity and different amplitudes, as in the cases (e) and (f). Three of these epochs (b, c and e) had a range of the acceleration modulus lower than the threshold of 30 × 10^−3^ g, and were therefore classified as IMA (see Methods). The remaining epochs had an acceleration slightly greater than this threshold and were therefore not classified as IMA.

In general, the PSD was shifted towards the left (low frequencies) with respect to that obtained from voluntary contractions in healthy subjects. In our data, the PSD mean frequency ranged between 44 Hz and 60 Hz, whereas in the voluntary contractions of the biceps of healthy subjects it is in the order of 70–80 Hz [26]. This is likely due to the low-pass filtering effect of the large electrode size and interelectrode distance adopted in this work [19].

### 3.4. Feasibility of IMA Detection 

Recorded hours, signal quality, sEMG and IMA duration are shown in Table 1 for the 10 subjects. 

EMG activity was found in 128.2 h, i.e., in 37% of recordings, either with or without arm movement. Absence of arm movement was found in 252 out of 350.5 h of reliable data (72%). The mean (standard deviation) of the range of the acceleration modulus was 14 (5) × 10^−3^ g. The remaining epochs, classified as “with movement”, presented a range of the acceleration modulus of 109 (127) × 10^−3^ g. IMA was found in all subjects (see Table 1). In total, IMA was found in 85.9 h, resulting in 25% of the data being reliable (Table 1). The large variability in IMA duration among subjects is clearly portrayed.

**Figure 5:**
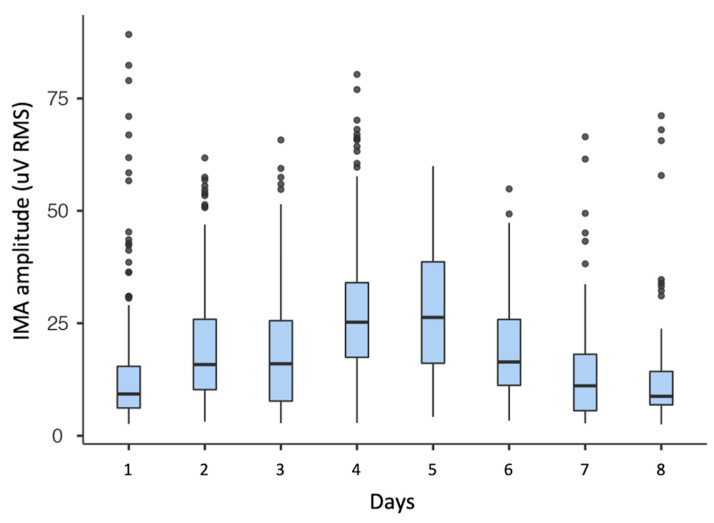
Boxplot of IMA RMS amplitude of one subject over the course of eight days of consecutive weeks of measurements.

In the sample, IMA RMS amplitude was on average 21 (16) mV and varied between 3 and 153 mV. Peak-to-peak IMA amplitude was on average 225 (171) mV and varied between 21 and 1894 mV. IMA variability within a single day and between days is shown for one subject (ID 008-Xr) in Figure 5. The single day variability is shown in the spread of a single box-plot. The between days variability is clearly visible when comparing consecutive boxplots. 

**Figure 6:**
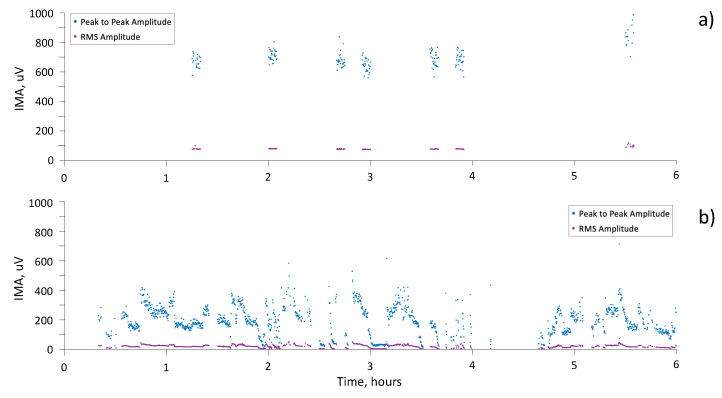
Peak-to-peak (blue dots) and RMS (purple dots) amplitude of IMA during 6 h of recording for (**a**) subject 002-E and (**b**) subject 001-Br.

Finally, Figure 6 shows the results of six hours of monitoring, with IMA detection, for two subjects. Both peak-to-peak and RMS amplitude are reported.

## 4. Discussion

In this study, we tested the possibility of using a commercially available device for sEMG with wearable probes and onboard memory to detect and monitor IMA in acute neurological patients at their bedside, where large EMI can be present, and with no modifications to the patients’ care, nursing activities and physiotherapy. To our knowledge, this was the first attempt to continuously collect hours of data from patients in these conditions.

The study’s results proved the in-field feasibility of using general-purpose sEMG sensors. Data were reliable in more than 80% of the 429 h of recordings and data quality was so good that even single MUAP trains were clearly recognized (see Figure 4). It is noteworthy that these results were obtained by using a pair of disposable electrodes using single differential configuration. 

As pointed out in the review by Vavrinsky et al. on modern multi-sensor Holters, the possibility of recording huge amounts of data in environments other than measuring laboratories presents issues in terms of the reliability, quality, and quantity of collected data [27]. Our study addressed all three issues.

EMIs produced by life-supporting devices (nutrition, hydration, tracheostomy vacuum cleaners) and by anti-decubitus air mattresses were the main cause of loss of data. These led to very large periodical interferences in the sEMG signal (see Figure 2). On the one hand, EMI-corrupted data were clearly unreliable, and filtering could be risky, as in the case of saturation. On the other hand, having such large amplitude, EMIs were clearly recognizable based on either their amplitude or their spectral characteristics. In this study, we decided to discard these epochs rather than filtering them to avoid any risk of false-positive detection of MUAP-like shapes that might result after the filtering procedure. When compared to the total duration of the acquisitions, EMIs were present for limited time intervals and their presence did not compromise the detection of the presence of IMA in the sample patients.

Data were also not reliable during physiotherapy, including arm mobilization, because of large motion artifacts and excessive probe and electrode movement (see Figure 3d). Should these data be of interest, as in the assessment of spasticity elicited by the externally imposed muscle stretch [28], a better adhesion of the probe to the skin would be necessary.

Uncorrupted data were further checked both in the time and the frequency domains to prevent any exceedingly low amplitude MUAP-like shapes to be classified as muscle activity—see description in the methods section. This approach was selected to minimize the risk of false-positive IMA detection, even though we acknowledge the possibility of having possible false-negative classifications. A further criterion to ensure IMA detection was the inclusion of patients with only complete UL plegia, who exhibited no traces of voluntary arm and hand activity. This condition was verified before each acquisition and the onset of traces of movement was the main reason of patient drop-out.

sEMG activity was found in all 10 patients, with a great variability among subjects in terms of duration. The onboard accelerometer allowed us to separate IMA from other forms of overactivity, based on the absence of movement. It is noteworthy that IMA was found in all 10 patients, despite their UL plegia. Hence, IMA can be present in acute stroke patients despite their clinical classification of plegia and this can be measured and monitored using wearable sEMG probes.

In four of the ten patients, IMA was present only between 1% and 6% of the overall recordings. Conversely, for two patients, IMA was found in 49% and 66% of the recorded time, respectively (see Table 1). This meant that these patients’ BB was active for long periods of time while at rest.

IMA was often present in the form of very long-lasting trains of one or a few MUs, insufficient to determine a mechanical effect, as in Figure 4a–c. As a result, IMA was characterized by low or exceedingly low RMS levels with much higher peak-to-peak amplitudes. The presence of few long-lasting trains of MUs is in line with the results obtained by both Campanini and colleagues in acute patients with UMNL evaluated in similar conditions using a linear array of electrodes [7], and by Mottram et al. and Forman et al. in chronic patients [8,10].

IMA consisting of MUAP trains could have been triggered by a tonic stretch of the muscle at rest, even when the patient is bedridden, depending on the arm position and the elbow angle. This is consistent with the relative inability to rest a muscle that is “responsive to the degree and duration of the tonic stretch imposed on the muscle” and is one of the consequences of UMNL [2]. Moreover, continuous activity in response to tonic stretches has been described as a trigger that supports the insurgence of muscle overactivity [2]. IMA consisting of MUAP trains was found in the BB muscle of chronic stroke patients, following a stimulus, while no spontaneous firing was recorded in the matched control muscle (the contralateral UL) neither at rest nor following voluntary contractions [10]. In healthy subjects, relaxed muscles show no trace of myoelectric activity [15]. Similarly, in healthy subjects, no activity is evoked by slow or tonic stretches, unless the stretch is rapid enough to trigger a reflex response [15]. In the BB of healthy subjects, a pattern of long-lasting trains of one or a few motor units can be induced when holding a light weight with their arm fully extended, since a mild tonic stretch is applied to the muscle–tendon unit.

This study found that muscle activity was also characterized by a series of mid-amplitude sEMG interferential signals. This resulted in higher RMS levels, higher peak-to-peak amplitudes and higher spectrum mean frequencies. Higher amplitudes might have been caused by several factors such as greater recruitment, particularly of superficial MUs recruited [29], or a reduced amount of fat layers between muscle and skin [30]. In healthy subjects, such amplitudes are commonly associated with a certain degree of mechanical effect on elbow flexion. In our data, we found cases with relatively high sEMG amplitudes and with no or minimum acceleration recorded by the probe. This peculiar pattern may be explained by the presence of antagonistic muscle co-activation. In future studies, this phenomenon should be investigated by placing a probe on the triceps. 

In the literature, IMA has been previously detected in chronic stroke patients using bipolar sEMG. In a study by Trompetto et al., IMA was found in the flexor carpi radialis muscle at rest in 17 patients, out of the 23 evaluated chronic stroke patients [9]. In a study by Forman et al., eight out of eight chronic stroke patients presented IMA in the biceps brachii when the muscle was extended [8]. This phenomenon was seen in chronic patients, who presented an increased resistance to passive movement and a clinically assessed spasticity (modified Ashworth scale score ≥ 1) [8]. A remarkable result of our study is that we found sustained IMA in muscles with complete plegia. This finding supports the statement by Trompetto et al. that spastic dystonia cannot be recognized on the sole basis of clinical examination [9] and requires sEMG assessment.

In all previous studies, the duration of IMA recordings was limited to just a few minutes. The presence of IMA has been assessed by visual inspection of sEMG traces [8,9,10]; a procedure that cannot be obviously applied to long-lasting acquisitions. The method implemented in this study allowed us to detect IMA based on a steady presence of MUAPs, in accordance with the methodology described by Forman et al. [8]. This method has the advantage of being automatic. In two studies, Wislow and Mummidisetty have successfully employed automatic recognition techniques of specific muscle behavior alterations (clones and spasms), caused by lesions in the central and peripheral nervous system, using long-lasting sEMG acquisitions, supporting its use in the clinical field [31,32].

Our method exploits the a priori information regarding MUAP morphology for detecting muscle activity [24], instead of modelling the sEMG signal as a band-filtered and amplitude modulated Gaussian noise [33,34]. The latter approach is adequate when modeling contractions leading to interferential sEMG but does not apply to IMA when only a few motor units are present.

Since single MUAPs can be identified, an analysis of firing rate, inter-spike interval, synchronization and coherence between MUAP trains can also be carried out to support the investigation of the pathophysiological mechanisms underlying IMA [7,8]. The presence of significant coherence between MUAP trains has been suggested as a possible indicator of a central origin (a common driving source of excitation of multiple motor neurons from descending pathways or from regional interneurons), whereas a consistent lack of coherence would support the hypothesis of an intrinsic cellular mechanism of hyperexcitability [10]. In addition, HDsEMG could be used in physiological research to assess motor unit properties, such as their conduction velocity, which cannot be obtained by bipolar detection, as in a study by Campanini et al. [7].

From a clinical point of view, it may be of great interest to monitor IMA insurgence and to follow its evolution over time, as well as to associate it with clinical variables, such as the reduction in range of motion, and with other clinical signs of muscular overactivity [35]. Moreover, the variability of IMA both within and between days found in this study (see Figure 5) suggests the need for long-term measurements to accurately describe a patient’s IMA, rather than taking into consideration just a few minutes of laboratory measurements [8,9]. Further studies should address this topic in order to determine the optimal length of the observation period and the optimal settings for an accurate assessment.

It may be interesting, for physiotherapists, to determine, when possible and in real time, IMA variations consequent to the shifting of patients’ postures (from supine to a seated position), different seated postures (with aids and supports), or from a sitting to a standing position. In addition, the amount of IMA can be used as an outcome in the assessment of the effectiveness of physiotherapy treatments aimed to maintain muscle length and extensibility, such as stretching protocols and manual therapy treatments [14,16,36,37].

### Limitations and Future Developments

This study was primarily focused on the feasibility of in-field IMA measurements using wearable probes. Hence, data were not analyzed and discussed from a clinical point of view. We plan to address this topic in a future study by investigating the relationship between IMA’s characteristics and the patient’s clinical history and their current status, as well as IMA’s possible predictive ability in the development of muscle overactivity and joint retractions.

## 5. Conclusions

Commercially available wearable probes with embedded inertial sensors and onboard memory can be used to monitor IMA in acute patients with plegia subsequent to UMNL during their stay in acute rehabilitation wards. IMA can be detected using a pair of disposable surface electrodes in a single differential configuration and can be characterized in terms of amplitude, duration, and within a day and between days variability. The opportunity of easily measuring IMA in acute inpatients with UMNL can have a huge impact on the management of their rehabilitation. 

## Figures and Tables

**Table 1:** Recorded hours, signal quality, sEMG duration and involuntary muscle activity (IMA) duration. sEMG refers to any presence of muscle activity, irrespective of the amount of upper limb acceleration recorded by the probe; IMA refers only to the presence of muscle activity without upper limb acceleration, with the patient at rest.

ID	Days of Recording	Recorded Data (Hours)	Reliable Data (Hours; % of Recorded Time)	sEMG Duration, (Hours; % of Reliable Data)	IMA Duration, (Hours; % of Reliable Data)
001-Br	6	35.2	26.1; 74%	16.6; 64%	12.7; 49%
001-Bl	6	35.2	32.1; 91%	20.7; 65%	15.2; 47%
002-E	10	59.9	41.5; 69%	22.5; 54%	12.8; 31%
003-Hr	1	5.0	4.4; 87%	3.0; 68%	2.9; 66%
003-Hl	1	5.0	4.4; 87%	2.9; 67%	2.8; 65%
006-R	11	61.8	54.5; 88%	16.4; 30%	8.8; 16%
007-U	2	8.0	7.8; 97%	0.4; 5%	0.2; 2%
008-Xr	8	46.8	40.9; 87%	18.2; 45%	12.3; 30%
008-Xl	8	46.8	42.0; 90%	19.9; 47%	12.9; 31%
00A-3	2	12.7	11.5; 90%	0.6; 5%	0.3; 2%
00H-P	5	30.1	21.0; 70%	4.7; 22%	3.5; 17%
00I-S	10	59.6	42.4; 71%	1.3; 3%	0.4; 1%
00J-Vr	2	11.7	11.4; 98%	0.7; 6%	0.7; 6%
00J-Vl	2	11.7	10.7; 92%	0.3; 3%	0.3; 3%
**Total**		**429**	**350.5; 82**	**128.2; 37%**	**85.9; 25%**

## Data Availability

The data presented in this study are available on request from the corresponding author.
